# Intracerebroventricular streptozotocin-induced animal model of Alzheimer’s disease: revealing dose optimization, administration regimen, and molecular pathways

**DOI:** 10.1186/s42826-026-00278-6

**Published:** 2026-04-13

**Authors:** Sagar A. More, Radhika N. Mundke, Yogeeta O. Agrawal, Sanjay N. Awathale, Sameer N. Goyal, Kartik T. Nakhate, Sumit S. Rathod

**Affiliations:** 1 Department of Pharmacology, SVKM NMIMS Global University School of Pharmacy and Technology Management, Dhule, Maharashtra 424001 India; 2 Department of Pharmaceutics, SVKM NMIMS Global University School of Pharmacy and Technology Management, Dhule, Maharashtra 424001 India

**Keywords:** Streptozotocin, Intracerebroventricular, Dosage optimization, Alzheimer’s disease, Neuroinflammation, Oxidative stress

## Abstract

Streptozotocin (STZ) is a commonly administered chemical via the intracerebroventricular (ICV) route in rodents to induce Alzheimer’s disease (AD)-like symptoms. Unfortunately, available research articles from different laboratories suggest inconsistency in doses, administration schedules, and target brain regions. For instance, ICV dose varies from 1.5 to 5 mg/kg either unilaterally or bilaterally, for 7 days to 21 days, as a single or multiple injections. Therefore, it is challenging for novice investigators to select the optimal doses, treatment durations, and targeted molecular pathways while employing STZ to induce AD-like conditions in experimental animals. In this background, the information related to the STZ-induced animal model of AD should be available on a single platform to aid researchers in selecting the suitable doses and regimens that suit their research objectives. The literature search was carried out by employing search engines such as PubMed and Google Scholar with keywords such as streptozotocin, intracerebroventricular, dosage optimization, Alzheimer’s disease, neuroinflammation, and oxidative stress. The present review highlights the articles published up to May 2025, with predefined inclusion (ICV-STZ model, cognitive/biochemical outcomes) and exclusion criteria (non-peer-reviewed papers, studies lacking behavioral or molecular endpoints, and studies older than 20 years). We have attempted to present the optimal dose, administration regimen, and biological process for inducing ICV-STZ AD models based on their advantages and limitations. According to the comparative quantitative evaluation of preclinical investigations, bilateral ICV administration of STZ at a cumulative dose of 3 mg/kg (1.5 mg/kg on days 1 and 3) displays the most reliable and physiologically relevant regimen. Studies also demonstrated that between 14 and 21 days after injection, this regimen reliably causes oxidative stress, neuroinflammation, cholinergic dysfunction, and progressive cognitive impairments, offering a balanced picture of physiological, histological, and behavioral alterations. However, unlike transgenic models, the ICV-STZ paradigm generally fails to develop extensive amyloid-beta plaque deposition or well-formed neurofibrillary tangles. Hence, more quantitative investigations are necessary to validate this optimization.

**Graphical Abstract:**

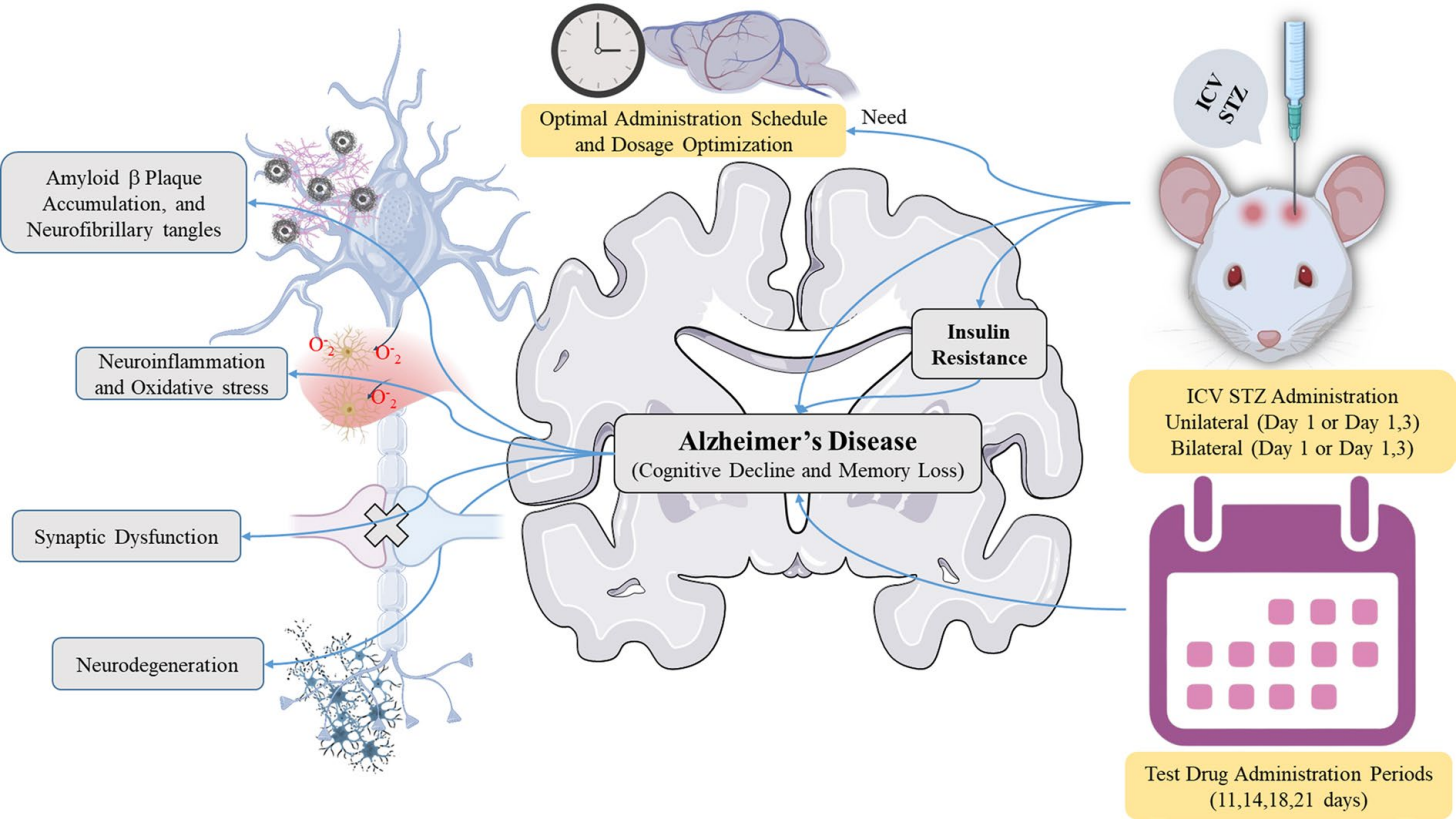

## Background

A wide range of disorders can be included in dementia, which is defined by impaired cognitive function that makes daily activities more challenging. Alzheimer’s disease (AD) is the most prevalent type, affecting the largest number of cases in those 65 years of age and older. Memory, language, thinking, and judgment are cognitive and behavioral abilities that gradually deteriorate with AD. While there is presently no known cure for AD, several therapies may be useful to improve several symptoms [1]. To date, there are 6.7 million American citizens aged 65 and older who are suffering from AD. This figure is estimated to increase to 13.8 million by 2060. AD was ranked as the sixth most prevalent cause of death in the years 2020–2021 [2]. In the later stages of AD, patients experience profound memory loss, hallucinations, confusion, and dependence on others for daily tasks. Eventually, mortality occurs from complications such as respiratory issues [3], infection, or fasting [4]. AD is characterized by amyloid-beta (Aβ) plaques, neurofibrillary tangles (NFTs), gliosis, and neuronal loss [5–8], as well as cerebrovascular amyloidosis, inflammation, and significant synaptic alterations [9–11].

Professor Sigfried Hoyer is credited as the originator of the intracerebroventricular-streptozotocin (ICV-STZ) model. His initial contributions to this field were consolidated in a scholarly publication dating back to 1994, where he delineated the foundational principles and concepts of the model [12]. ICV-STZ is a non-transgenic metabolic model for sporadic Alzheimer’s disease (sAD) [13]. Hoyer’s line of reasoning began with the observation that in sAD, there is a decline in both brain oxygen and glucose consumption, with the former being less affected. This discrepancy led to the hypothesis that early-stage sAD primarily involves impaired control over cerebral glucose metabolism due to a failure in cerebral insulin receptor signal transduction. Furthermore, studies on adult male Wistar rats demonstrated that giving 80 mU of insulin directly into the brain increased the activity of two important enzymes involved in glucose breakdown, hexokinase and phosphofructokinase, by approximately 20%. This implies that insulin plays a part in controlling how the brain metabolizes glucose. This finding implied that any disruption in insulin signal transduction could significantly affect brain glucose metabolism [14–16]. The subsequent action involved the ICV administration of STZ to disrupt the regulation of cerebral glucose and energy metabolism.

The ICV-STZ paradigm continues to gain momentum in neurodegenerative disease research, notably for sAD, which accounts for more than 90% of all AD patients [17, 18]. In contrast to transgenic animals, which predominantly replicate familial mutations, ICV-STZ causes a multifactorial disease that includes cognitive decline, insulin resistance, oxidative stress, and cholinergic impairments, all of which are major hallmarks of sAD [19, 20]. STZ, a glucosamine-nitrosourea compound injected ICV, inhibits cerebral glucose metabolism and insulin signaling without triggering systemic toxicity, preferentially mimicking central insulin resistance, a putative early cause of AD pathophysiology [21, 22]. Furthermore, findings also recognized AD as “type 3 diabetes” [23, 24]. The ICV-STZ model uniquely represents the neuro-metabolic foundations of AD, making it particularly useful for evaluating insulin-sensitizing interventions, metabolic modulators, and oxidative stress-targeting drug therapies. While multiple studies have identified abnormalities in insulin signaling and increased oxidative stress, there is a lack of consistent characterization of the altered cellular pathways, limiting mechanistic understanding.

The ICV-STZ model is recognized as one of the most useful non-transgenic approaches for replicating early-stage sAD [25]. Low-dose STZ has been shown to inhibit central insulin signaling, overstimulate GSK-3β, alter glucose metabolism, and cause oxidative and proinflammatory responses, all of which are linked to the pathophysiology of sAD [26, 27]. These basic abnormalities mimic the biochemical and neurological components of the human disease. The model accurately replicates key phenotypic features of sAD, such as gradual learning and memory deficits, diminished cholinergic activity, impaired mitochondrial function, synaptic decline, elevated soluble Aβ species, and early hyperphosphorylation of Tau [25, 28, 29]. However, in line with its limited validity, the model does not generally produce entire amyloid plaques or neurofibrillary tangles, indicating early rather than late AD disease. These validity categories warrant the adoption of the ICV-STZ approach for investigating the molecular basis and therapeutic strategies relevant to early sAD.

The present review addresses knowledge gaps by methodically collecting current knowledge on dose-response associations, protocol variation, and molecular disturbances related to the ICV-STZ model. Through closely examining reported outcomes across various dosage paradigms and administration regimens, the study provides a framework for identifying context-appropriate parameters adapted to specific research objectives. Furthermore, it incorporates molecular study data to identify repeating patterns and relevant biomarkers. Importantly, this study does not seek to replace current models, but rather to improve the scientific rigor by which the ICV-STZ framework has been executed. The present review also contributes to a more repeatable and mechanistically grounded implementation of this model by revealing gaps in technique and interpretation, as well as providing avenues toward harmonization. This is essential not only for validating therapy targets but also for enhancing our comprehension of sAD pathology at the metabolic and molecular levels. In the subsequent section, some hypotheses are presented to elucidate neurological deterioration and the basis of cognitive pathology.

## Overview of Alzheimer’s disease pathology and its molecular hallmarks

**Fig. 1:**
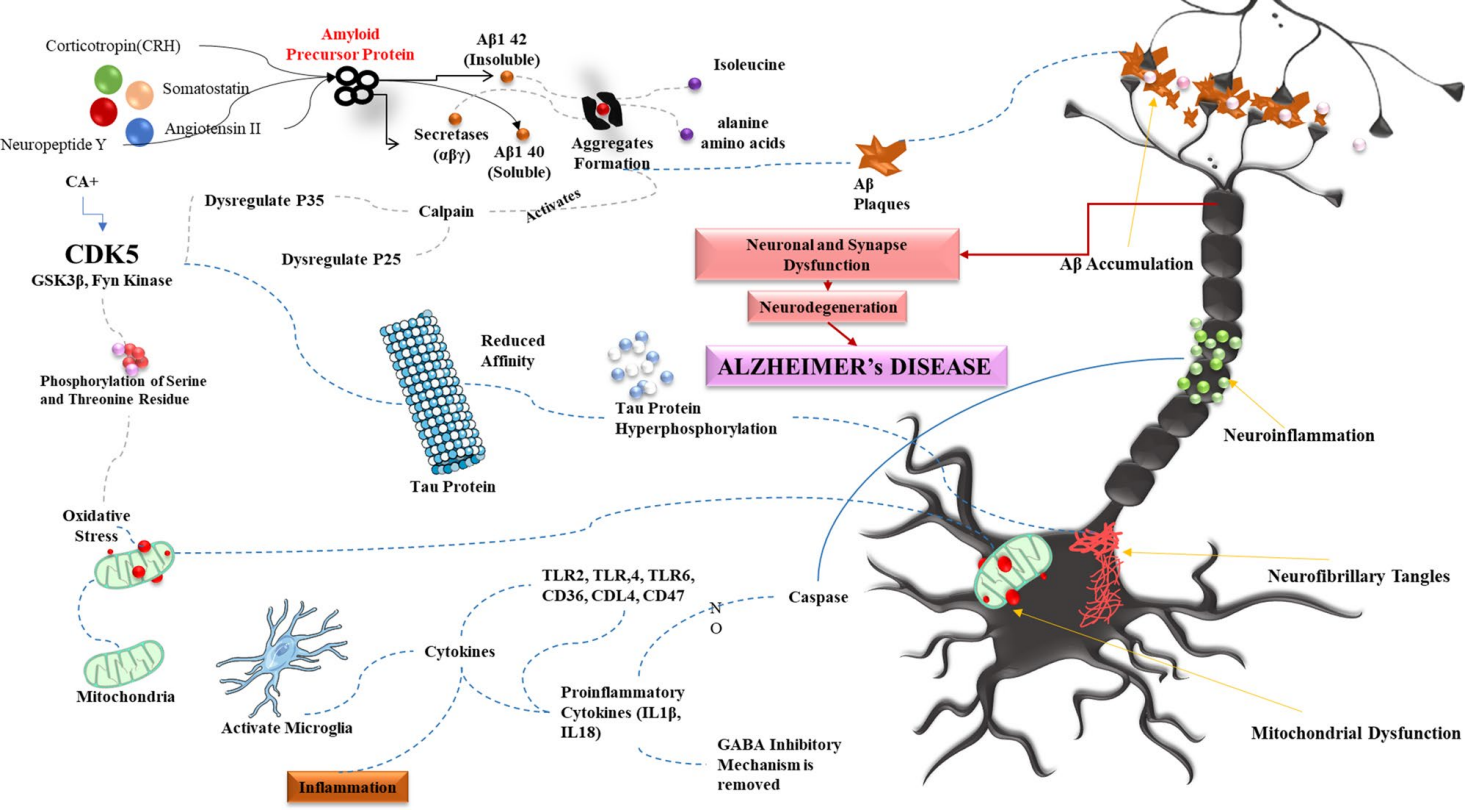
Molecular pathways involved in Alzheimer’s disease pathology

AD is an intricate brain disease characterized by the accumulation of senile plaque and tau-induced nerve injury. Apart from amyloid and tau, researchers have discovered numerous additional alterations and disturbances in the cellular pathways, demonstrating that AD development involves multiple triggers and pathways. The presence of Aβ plaques in various brain regions triggers an immune response as the brain perceives them as foreign objects. This response activates microglia and prompts the release of cytokines, culminating in cell death and neurodegeneration. Aβ plaques are made up of Aβ peptides that come from the amyloid precursor protein (APP). These peptides are created when secretases (specifically α, β, and γ types) cut the APP through enzymatic processes [30]. Neurofibrillary degeneration involves tau proteins, which are essential microtubule-associated proteins within neurons [31]. These proteins have a domain that binds to microtubules, aiding in their assembly and stability, thus maintaining cytoskeletal integrity. This binding process is regulated by the phosphorylation of serine/threonine residues mediated by various kinases, such as Fyn Kinase, glycogen synthase kinase-3β (GSK-3β), and cyclin-dependent kinase-5 (CDK5) [32, 33]. Hyperphosphorylation leads to a decreased affinity of tau proteins for microtubules. The hyperphosphorylated tau subsequently forms NFTs and accumulates in the cytosol, unable to fulfill its role in maintaining cellular structure [32, 34] Fig. 1.

The cognitive process involves the cholinergic system, and its dysfunction contributes to various forms of dementia, including AD. Previous studies reported that the selective accumulation of amyloid plaques and NFTs in cholinergic neurons within the nucleus basalis of Meynert (nbM) serves as a topographic marker for degenerating cell clusters in AD [35, 36]. This degeneration is exacerbated by the initiation of pro-inflammatory processes mentioned earlier, which further impair cognitive function [37]. Neuroinflammation is an important factor contributing to the progression of AD. Acute inflammation can prevent brain damage induced by Aβ plaque [38]. Aβ deposits trigger toll-like receptors (TLRs) and co-receptors like cluster of differentiation 36 (CD36), CD14, and CD47 on microglia, leading to the production of pro-inflammatory cytokines such as interleukin-1β (IL-1β) and interleukin-18 (IL-18) by caspase-1 or caspase-8 activation [39, 40]. This dysfunction, coupled with the overactivity of GSK-3β as a result of oxidative stress, results in the disruption of mitochondrial permeability, which, in turn, enhances reactive oxygen species (ROS) production [41, 42]. Genetic and environmental variables, as well as reduced metabolic activity, all contribute to the initiation and progression of AD [43]. Recent investigations have demonstrated the connection between AD and systemic metabolic alterations that include glucose and oxygen hypometabolism [44], lipid peroxidation [45], irregularities of Aβ metabolism and transport [46], and deficiency or excess of biogenic metallic components [47, 48].

**Fig. 2:**
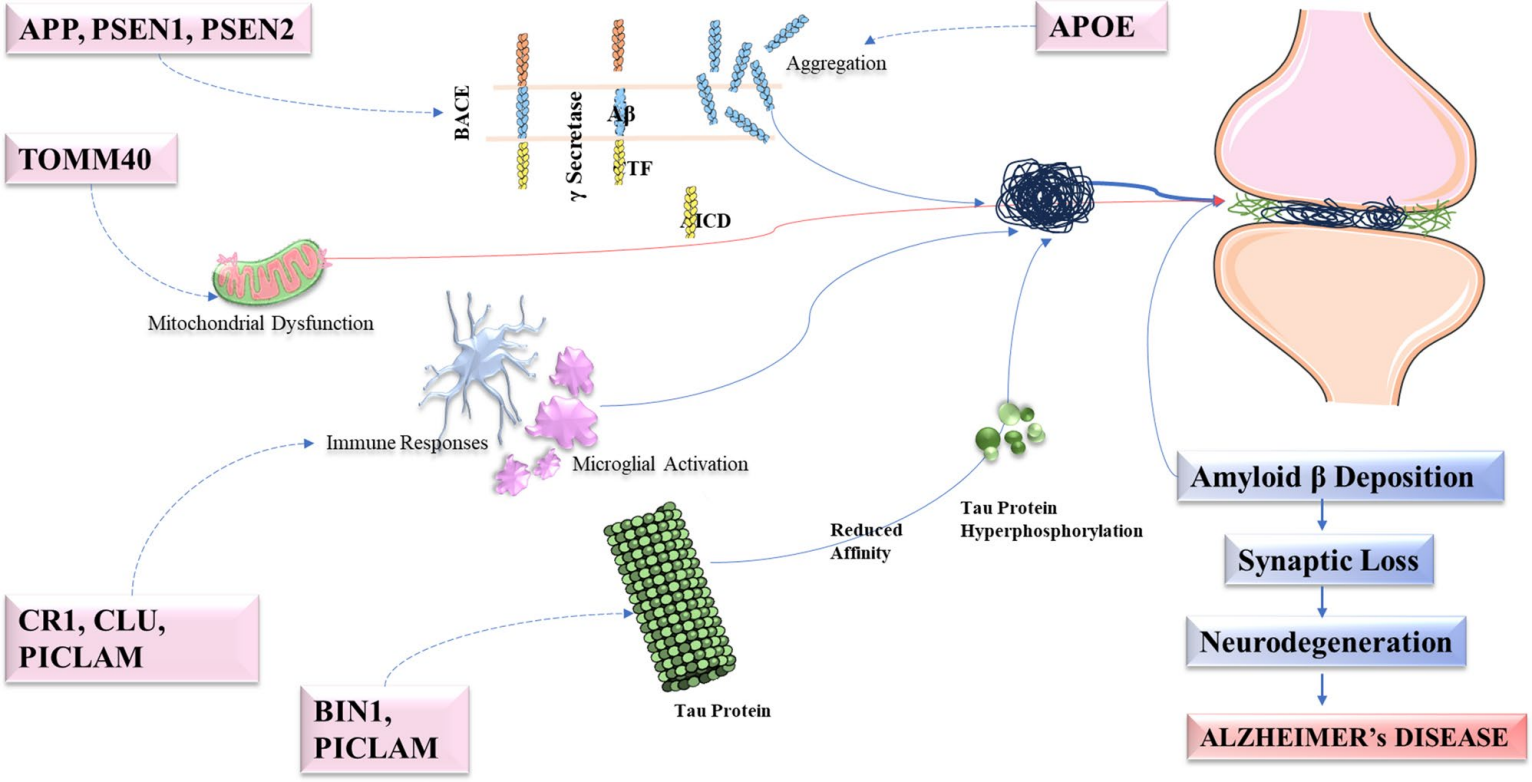
Genetic involvement in Alzheimer’s disease

AD is a complex disease influenced by various genetic factors. Mutations to the *APP*, *PSEN1*, and *PSEN2* genes impair the normal processing of Aβ, resulting in autosomal dominant AD [49]. These genes carry rare variants that significantly increase the risk of late-onset AD in families. Modulation of *APOE* and other genetic locations on age at onset in families with PS2 mutation [50]. *ADAM10* produces primary α-secretase in the brain, which is the principal enzyme responsible for preventing the formation of Aβ through cleavage within the Aβ domain of APP [51]. However, late-onset AD-linked mutations can directly inactivate this α-cleavage function of *ADAM10*. These mutations are shown to impair α-secretase cleavage of APP both in vitro and in vivo, underscoring the contribution of *ADAM10* dysfunction to late-onset AD pathology [52]. Clusterin (*CLU*) plays a role in transporting Aβ from the bloodstream to the brain and in the fibrillization of Aβ. Understanding this intricate genetic network not only sheds light on AD’s mechanisms but also holds promise for targeted therapeutic interventions Fig. 2.

## Rationale for intracerebroventricular streptozotocin-induced sporadic Alzheimer’s disease-like conditions

STZ is particularly detrimental to insulin-producing pancreatic beta cells, as evidenced by its cellular absorption via the low-affinity glucose transporter 2 (GLUT2) protein in their cell membranes [53]. STZ-associated cytotoxicity is mostly due to DNA alkylation, which causes cellular necrosis. In addition to pancreatic beta cells, systemically delivered STZ can harm other organs that produce GLUT2, such as the kidney and liver; however, the brain is not directly harmed since the blood-brain barrier excludes this transporter protein [54]. However, single or repeated ICV-STZ injections can impair cerebral glucose absorption over time and induce a variety of additional consequences that mirror the molecular, clinical, and behavioral characteristics of AD. Recognizing that glucose hypometabolism is an early and consistent symptom of AD, and AD brains display signs of altered insulin signaling, some researchers use ICV-STZ injections as a non-transgenic model of the disease for preclinical testing of drug therapies for AD.

Previously reported studies aimed to explore potential alterations in cerebral glucose utilization following ICV-STZ administration at a sub-diabetogenic dose (1.5 mg/kg body weight) [55]. These studies, along with similar ones, have shown that injecting STZ into the lateral ventricles of rats decreases the amount of glucose their brains use, particularly in areas such as the frontal and parietal cortex. This leads to lower levels of Adenosine triphosphate (ATP) and phosphocreatine, as well as a decrease in the ratio of ATP to ADP and the overall energy level in the brain’s cortex [56]. Moreover, researchers noted several other changes in brain function and behavior. In a separate study, it was proposed that giving three ICV-STZ injections may inhibit cerebral insulin receptors, although a mechanistic explanation for this phenomenon was not provided [15].

Earlier data suggested that the ICV-STZ injection rat model closely mirrors human AD patients in terms of both brain metabolic and behavioral disruptions. Recently, it was elaborated upon, asserting that both sAD and the ICV-STZ-induced model share characteristics of insulin resistance within the brain, termed an Insulin Resistant Brain State (IRBS). This suggests that ICV-STZ injections could be acknowledged as a non-transgenic preclinical model for AD [13]. The precise definition of IRBS remains elusive, as noted in a recent article. Previous discussions on IRBS as a potential cause of AD elucidate its functional resemblance to the pathophysiology of type II diabetes mellitus. However, to prevent confusion, authors suggest using the term IRBS rather than “diabetes mellitus type II confined to the brain” in the context of sAD. Previous study reveals that STZ suppresses insulin receptor substrate (IRS)-1 and consequent Akt phosphorylation, resulting in reduced glucose absorption and enhanced tau phosphorylation via overexpression of GSK-3β [57, 58].

The process of rats developing AD-like signs of brain metabolic and behavioral changes after one or two ICV-STZ injections is seen as a dynamic journey. Initially, studies mainly looked at how ICV-STZ affected brain functions and behaviors over a short span, usually a few weeks. However, a recent study by Hoyer’s team extended this observation period with ICV-STZ rats, revealing the presence of Aβ deposits in the linings of brain blood vessels and cortex, which they detected using staining techniques like thioflavin-S and Congo red, along with immunohistochemical methods [59]. According to some recent findings, cognitive and neurochemical changes induced by ICV-STZ injection follow a distinct time-dependent pattern, characterized by three phases.

## Importance of determining the optimal dose, administration schedule, and underlying molecular mechanisms

The optimal dosage, administration schedule, and the underlying molecular mechanisms of ICV-STZ administration need to be determined in developing effective treatments against AD. Rodent STZ-induced models have been widely used in the study of AD pathology. The expression of brain metabolic disturbances and behavioral changes in the models was similar to that seen in AD [60]. The ICV-STZ rat model provides a very close resemblance to the human sAD process; hence, it becomes of great value as a preclinical model for the study of disease [18]. Studies have revealed that ICV-STZ administration induces neurodegenerative changes similar to those seen in diabetes-related AD, thus underscoring the need to study the molecular mechanisms and possible treatments [61]. Furthermore, STZ administration in rodents has been shown to inhibit brain mitochondrial activity, possibly mimicking the pathogenesis of sAD [62]. The infusion of STZ in sub-diabetogenic doses also induces changes in the brain’s glucose metabolism, a hallmark of sAD [63].

Among the major goals of this study was to determine STZ effects on brain insulin system dysfunction and tau protein hyperphosphorylation, concerning an understanding of AD pathogenesis. Parameters of ICV-STZ administration and molecular mechanisms involved need to be determined, and possible interventions explored, to the advancement of knowledge in understanding and treating AD. ICV-STZ models offer meaningful platforms for the study of the pathophysiology of AD and the testing of new treatments [13, 64]. Several neurochemical and neuropathological changes after ICV-STZ injections have been documented by several researchers. Some of these results are consistent with Hoyer’s findings, and all of them confirm that the metabolic lesions produced by ICV-STZ are global, that is, concerning the whole brain, at least at some distance from the site of injection, and progressive [65, 66].

## Molecular basis of intracerebroventricular streptozotocin-induced Alzheimer’s disease model

### STZ-induced Alzheimer’s disease-like neurotoxicity

**Fig. 3:**
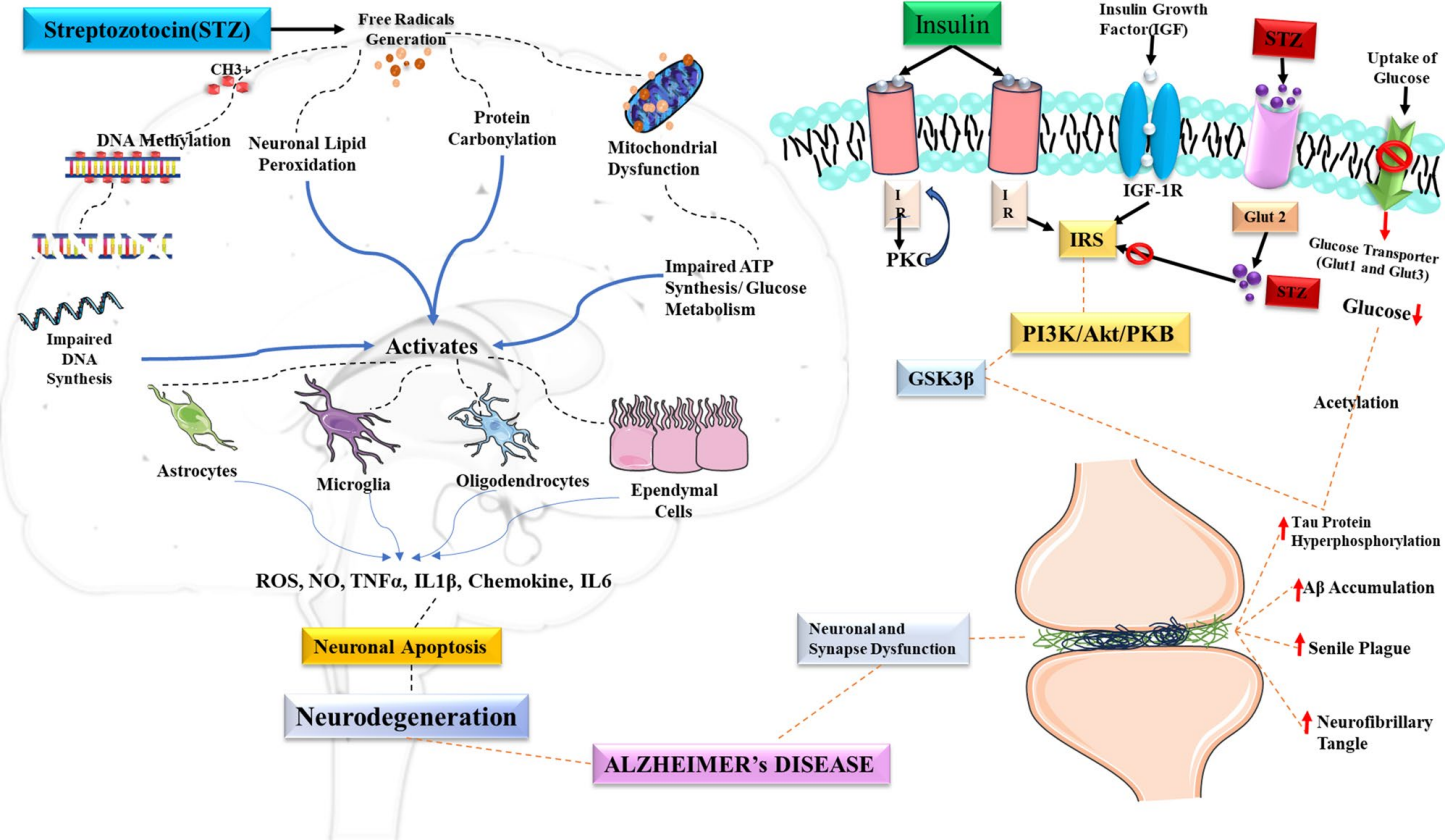
Mechanism of STZ-induced Alzheimer’s disease neurotoxicity

STZ used to induce diabetes in experimental models, acts by alkylating or methylating DNA. Even at sub-diabetic doses (3 mg/kg; ICV), it replicates AD pathology. Akin to other nitrosourea compounds, STZ generates carbonium ions, damaging DNA via methylation. Additionally, it leads to the death of neurons by causing DNA fragmentation, which is facilitated by its nitrosourea component and other processes. This damage to DNA activates poly ADP-ribosylation through poly ADP-ribose synthetase, reducing the levels of cellular NADH and ATP, which are essential for regulating cellular energy [67]. Previous studies have found that STZ increases the expression of glial fibrillary acidic protein (GFAP), CD11b, and TNF-α, suggesting increased glial activity and neuroinflammation [68, 69]. STZ also dramatically enhanced ROS, nitrite, and Ca^2+^ levels while decreasing mitochondrial function in synaptosomal preparation, demonstrating free radical production and excitotoxicity [70]. Increased expression and activity of Caspase-3 were also observed in STZ-treated rats, which specifies apoptotic cell death in the hippocampus and cortex [71]. STZ treatment showed decreased expression of postsynaptic markers CaMKIIα and PSD-95, while expression of presynaptic markers (synaptophysin and SNAP-25) remains unaltered, indicating selective postsynaptic neurotoxicity [72, 73] Fig. 3.

### Oxidative stress

Oxidative stress emerges as a crucial element in initiating cellular death in AD and serves as a mediator for various processes contributing to the pathophysiology of AD [74]. It triggers additional critical events such as mitochondrial dysfunction, protein aggregation, and glutamate excitotoxicity, which collectively contribute to neuronal death [70]. STZ administration to the brain results in significant reductions in glucose/energy metabolism and induces oxidative stress. In ICV-STZ mice, higher levels of malondialdehyde (MDA), decreased glutathione (GSH), and altered mitochondrial complex processes are commonly observed, indicating oxidative stress as a primary cause of neurodegeneration [75, 76]. Evidence has pointed out that STZ treatment generates free radicals, such as ROS and RNS, thereby increasing levels of oxido-nitrosative stress in rodents [77, 78]. Researchers have proved that STZ-induced oxidative stress normally leads to the enhanced level of MDA, which serves as a marker of lipid peroxidation and acts as an indicator of free radical generation, and glutathione, an endogenous antioxidant having free-radical-scavenging activity against oxidative and nitrosative stress. STZ treatment in the brain modifies markers of oxidative damage, such as thiobarbituric acid reactive substances, GSH, protein carbonylation, glutathione peroxidase, and GSH reductase related to decreased levels of ATP and mitochondrial dysfunction, which is correlated with cognitive impairments [79]. It was also noted that STZ administration significantly increases oxidative stress and neuroinflammation in rat brains by elevating MDA, TNF-α, and IL-1β levels, as well as GFAP, while reducing GSH levels. Furthermore, STZ induces NAD^+^ depletion and generates free radicals like hydrogen peroxide and nitric oxide [80–84]. STZ treatment induces oxidative and nitrosative stress, along with caspase activation in astrocytes, indicating its impact on glial cells. These findings collectively demonstrate that STZ triggers oxidative stress in the brain. However, oxidative stress typically correlates with brain inflammation, termed neuroinflammation, which could potentially exacerbate AD-like pathology [68].

### Neuroinflammation

Neuroinflammation is a key characteristic of AD and occurs before the development of plaques and tangles as the disease progresses [85, 86]. Neuroinflammation is closely linked to the development of AD, involving the activation of glial cells [87, 88]. The typical functions of glial cells can sometimes contribute to a more severe and prolonged neuroinflammatory cycle, which can worsen neurodegenerative diseases. In AD, the inflammatory components involve brain cells such as microglia and astrocytes. Inflammatory processes, including gliosis and the recruitment of microglia to affected areas, occur in the brain. Glial cells, such as microglia, astrocytes, and oligodendrocytes, support neurons and maintain their health [87, 89, 90]. In the case of activation, astrocytes and microglia release different products that become involved in inflammation and neuronal injury. These include cytokines TNF-α and IL-1β, but also anti-inflammatory cytokines such as IL-6, IL-10, and IL-4. It also releases chemokines such as interferon-gamma and macrophage inflammatory protein, along with free radicals such as nitric oxide and superoxide. Previous studies suggest that STZ-induced production of proinflammatory cytokines, IL-1β and TNF-α, leads to the production of neurotoxic oxygen- and nitrogen-based free radicals involved in neurodegeneration. STZ treatment enhances neuroinflammation, oxidative/nitrosative stress, and activation of caspases. It is these free radicals that ultimately lead to neuronal damage as their actions produce several inflammatory substances and other neurotoxic products in the process of neuroinflammation, which can potentially harm neuronal function [91, 92].

### Insulin and brain

Insulin, a hormone formed by pancreatic beta cells, regulates glucose uptake from the bloodstream. It plays a vital role in carbohydrate and fat metabolism and promotes glucose absorption. Glucose uptake occurs through the cell membrane glucose transporters. The insulin receptor on cell membranes facilitates glucose uptake by regulating protein kinase molecules internally. Brain insulin primarily originates from pancreatic beta cells, crossing the blood-brain barrier via a transporter distributed randomly throughout the brain [93]. Both immature and mature mammalian neuronal cells, besides pancreatic beta cells, demonstrate insulin synthesis and expression. Nerve endings have high concentrations of insulin receptors (IR) that specifically bind with insulin. Both neurons and glial cells in the brain can make insulin. Insulin’s special qualities help to reduce oxidative stress, improve how mitochondria create energy, and protect nerve functions when conditions are challenging. Moreover, insulin lowers the harmful effects of Aβ-peptides and tau hyperphosphorylation in rats, easing symptoms similar to those seen in AD [94–97]. STZ administration disrupts the insulin receptor gene expression and tyrosine kinase activity, thus disturbing the balance between phosphorylation and dephosphorylation of the IR subunit, finally leading to a dysfunction of the insulin system. The abnormalities may result in tau protein hyperphosphorylation and, therefore, also raise the possibility of a pathological link between impaired brain insulin function and tau hyperphosphorylation as a marker of sAD [98, 99].

### STZ-induced kinase activation

ICV-STZ exhibits AD-like clinical symptoms, including Aβ deposition, hyperphosphorylated tau protein, diminished insulin signaling, disturbed glucose absorption, oxidative stress, and cognitive impairment [100]. Disrupted PI3K/AKT/GSK-3 signal transmission, lower glucose metabolism, increased oxidative stress, and Aβ overload in ICV-induced memory impairment. STZ-induced inhibition of insulin receptor activity by inhibition of PI3K/AKT/GSK-3α, a downstream molecule of the insulin signaling pathway, is responsible for increasing Aβ expression and promoting memory loss in the rat brain [101]. GSK-3α, linked to PI3K and AKT pathways, triggers the formation of NFTs and senile plaque deposition, recognized as the primary lesions in AD [102]. Previous research on GSK-3β, an enzyme that’s less active in the IR-PI3K pathway, discovered differences in the phosphorylated form of the hippocampus. The Akt/PKB levels stayed the same after 4 weeks but dropped after 12 weeks following ICV-STZ treatment in the hippocampus, while they decreased after 4 weeks but rose after 12 weeks in the frontoparietal cortex. Phosphorylated GSK-3 levels went up after 1 month but decreased after 3 months of STZ treatment. These changes, such as increased GSK-3β activation and tau phosphorylation, may stem from disruptions in insulin and IGF signals in the brain/CNS. STZ also raised phosphorylated p38MAPK levels, suggesting the ROS-p38 MAPK pathway’s involvement, which is unrelated to blood sugar levels [21, 103, 104].

### Insulin, GSK-3β, and regulation of synapse function

IRs are found in astrocytes and neurons within the central nervous system [105]. IRs are highly expressed at synapses, particularly within the most plastic regions of the brain, including the cortex and hippocampus. This may indicate a putative role for insulin signaling within synaptic plasticity. Accumulating data indicate that insulin signaling plays a role in synaptic physiology. In the central nervous system, IRs are present in both astrocytes and neurons [106, 107]. IRs are found at connections between nerve cells, especially in busy brain regions like the cortex and hippocampus. This hints at a potential link between insulin activity and how our brain cells change and adapt. Earlier reported studies indicate that insulin activity plays a crucial role in how our brain connections work [108–110]. Insulin also has a role in causing long-term depression (LTD), which is a form of how brain connections change in AD. Insulin activates PI3K and protein kinase B (PKB), which leads to LTD happening in certain neurons in the hippocampus, influencing how brain connections adapt to activity, especially when NMDA receptors and the PI3K pathway are active [111].

Several molecules involved in insulin signaling are essential for synaptic plasticity, indicating their importance in learning and memory processes. It means that this activation of insulin receptors triggers the downstream PI3K/Akt pathway, which has already been known to play an important role in synaptic plasticity [112]. Akt deactivates GSK-3β by adding a phosphate group to the serine-9 residue. There are also findings indicating that GSK-3β interacts closely with GluR1/2 AMPA receptor subunits, and their function is vital for how brain connections change and improve. Turning off GSK-3β is important for triggering long-term potentiation (LTP), a process that strengthens synaptic connections [112–115]. STZ roots deficits in hippocampal synaptic transmission, and LTP was observed. The deterioration in LTP is connected with altered expression of NMDAR subunits [116, 117]. The decrease in LTP abilities was linked with alterations in how synaptic AMPARs are expressed and function, lower levels of brain-derived neurotrophic factor (BDNF), and changes in both presynaptic and postsynaptic membrane proteins [118, 119].

### STZ-induced neuropathological changes

STZ injections into the brains of rodents have been linked to specific changes in brain structure, noticeable as soon as one week after a single dose [120, 121]. STZ administration in the hippocampus leads to higher levels of GFAP, which indicates increased gliosis. This suggests that changes in hippocampal function due to STZ could lead to difficulties in memory [122, 123]. Histopathological studies have shown that STZ can lead to the selective loss of certain neurons. This occurs because STZ is neurotoxic to axons and myelin, resulting in the demyelination of neurons in brain areas linked to learning and memory, such as the hippocampus and periventricular areas [19]. Researchers found that STZ causes an increase in the expression of amyloidogenic proteins and boosts levels of APP, BACE-1, and Aβ1–42 in astrocytes. These changes are similar to the pathological features observed in the brains of patients with sAD [68]. Furthermore, scientists also noted that injecting STZ resulted in a notable enlargement of the Golgi apparatus. This enlargement might affect how beta-amyloid precursor proteins (β-APP) are broken down in the endoplasmic reticulum and Golgi complex, contributing to the build-up of these proteins in the brains of individuals with AD [124].

### STZ-induced amyloid-beta and tau hyperphosphorylation

Extracellular buildup of Aβ plaques mainly involves clumps of the harmful Aβ1–42 sequence. These clumps are formed naturally through the specific breakdown of the amyloid precursor protein (APP) [125]. The amyloid cascade theory suggests that the abnormal build-up of Aβ is the main trigger for both forms of AD, and all associated brain changes, like cell loss, inflammation, oxidative stress, neurotransmitter imbalances, and the resulting decline in cognitive abilities, are a result of the harmful effects of Aβ build-up [18]. Clinical findings indicate that alterations in insulin and insulin receptor signaling pathways in the brains of individuals with AD can affect the metabolism of APP and the build-up of Aβ, as well as the equilibrium between phosphorylated and non-phosphorylated tau proteins. Previous research revealed that treatment of STZ into the brain of rats affects insulin system activity and builds up hyperphosphorylated tau protein [126]. Tau protein normally helps stabilize and support microtubules in neurons, but when it becomes excessively phosphorylated, it takes on a harmful role that leads to neuronal death [62]. Findings demonstrate that injecting STZ into the brain increases tau protein and Aβ levels across the cerebral cortex and hippocampus after three weeks, based on immunohistochemical analysis [63, 127]. Additionally, STZ triggers the activation of several kinases by blocking the action of phosphatase (PP2A), which normally removes phosphate groups from these sites. Previous studies also revealed that STZ may trigger the formation of neurofibrillary tangles (NFTs), which are constituted of the build-up of overly phosphorylated tau protein within neurons [64]. The study found that ICV-STZ treatment consistently increases soluble Aβ compounds and hyperphosphorylated tau forms, indicating early-stage AD-like neurological dysfunction. Interestingly, these changes often occur in the absence of extrinsic amyloid-beta plaque development or mature neurofibrillary tangles, suggesting that the model replicates early molecular disruptions rather than complete structural markers of AD.

## Administration schedule of intracerebroventricular streptozotocin-induced Alzheimer’s disease-like conditions

Establishing an appropriate administration schedule for ICV-STZ injections is crucial for accurately modelling AD. Using ICV injection of STZ has become widely recognized as a model to study AD [76]. This method induces an insulin-resistant brain state, proposed as an experimental model of sAD [128]. Research has demonstrated that administering STZ through ICV leads to disruptions in insulin signaling, glucose transporter functions in the brain. Continuous long-term intranasal insulin in rat models with ICV-STZ-induced impairment revealed improvement in cognitive functions [129, 130]. STZ-administered ICV has been shown to result in long-lasting reductions in learning and memory abilities, as well as cerebral energy metabolism in rats [131]. This method induces AD-like changes in protein kinase B and GSK-3 in the brain [57, 132]. Chronic administration of pioglitazone attenuates the memory impairment induced by ICV-STZ injections. Moreover, ICV administration of STZ has been used as a rodent dementia model in a variety of dementia studies in animals [133].

## Impact of STZ injection frequency and duration on Alzheimer’s disease progression

The frequency and duration of AD-like symptoms after STZ administration via the ICV route have been documented in great detail. It is reported that one or more injections via the ICV route with STZ may induce permanent reductions in the quantity of glucose taken up by the brain and several AD-like alterations. These include progressive decline in memory, brain insulin resistance, decreased brain glucose utilization, synaptic function impairment, biochemical alterations of Aβ accumulation, and tau hyperphosphorylation [134]. Finally, it has been shown that memory deficits were detected at specific time points post-STZ injection at 14 and 21 days and thus represent cognitive dysfunction associated with AD progression [135]. It has been demonstrated that ICV-STZ at a dose of 3 mg/kg induces problems in cognitive and neuropsychiatric abilities through neuron and synapse damage, strongly related to alterations in neuronal function [29]. Respiratory dysfunction has also been described at rest and in response to hypoxia following ICV-STZ, showing more global physiological effects beyond just symptoms in the cognitive domain [136]. Respiratory dysfunction has also been described at rest and in response to hypoxia following ICV-STZ, showing more global physiological effects beyond just symptoms in the cognitive domain [136].

Concerning the frequency/duration of injection of STZ on AD, many animal models have been studied. One of the common techniques for inducing AD-like changes in rodents involves ICV injection of STZ into the ventricles of the brain. This led to problems in cognition, increased phosphorylation of tau, and accumulation of amyloid beta, amongst many other pathological traits associated with sAD [137]. The ICV injection of STZ has been shown to exaggerate AD-like changes in various mouse models, thus showing its role in the progression of the disease [25]. It has been further reported that ICV STZ injection causes synaptic downscaling, hippocampal atrophy, and ventricular enlargement in an animal model of cognitive dysfunction and anxiety [138]. Moreover, insulin responses, tau hyperphosphorylation, and neuroinflammation have been addressed after STZ injection in connection with impaired insulin function and early biochemical alterations in AD [139, 140].

## Importance of establishing an appropriate administration schedule

The usefulness and therapeutic significance of the ICV-STZ-induced AD model are significantly dependent on the optimal delivery schedule. This includes not only the dosage, but also the duration, period, and volume of doses. An appropriate dosage regimen is critical for simulating the progressive and multifaceted character of sAD, especially in terms of cognitive impairment, neural inflammation, insulin resistance, and cholinergic impairment [81, 141]. A precise dosing schedule confirms that the model is reproducible across various labs and experimental scenarios. It enables for controlled generation of neurodegenerative alterations that closely mirror the pathogenesis of late-onset AD, as compared to acute neurotoxic or nonspecific brain damage. Several studies suggest that single high-dose injections can cause prevalent damage to neurons and divergent consequences, while fractionated lower-dose regimens (e.g., 3 mg/kg STZ on two alternate days) can more consistently replicate early sAD-like pathology such as tau hyperphosphorylation, oxidative stress, and diminished synaptic plasticity [19, 103]. Furthermore, the time between STZ treatments increases the severity and development of cognitive impairments. A precise timetable allows for the chronicity necessary to imitate the gradual progression of AD. For example, delivering STZ on Days 1 and 3 creates an episodic interval that promotes prolonged neuroinflammatory transmission and insulin signaling imbalance, both of which are characteristics of sAD [76]. Significantly, the administration schedule influences the initiation of subsequent biochemical processes. Tailored regimens can specifically regulate important biomarkers, including GSK-3β, BACE-1, IRS-1, and inflammatory mediators, which are crucial for assessing treatments and understanding disease processes [142, 143]. Thus, a suitable delivery schedule is required not only to improve the model’s validity and reliability but also to guide preclinical drug development activities targeted at early detection and management of AD.

## Dose optimization of intracerebroventricular streptozotocin-induced Alzheimer’s disease-like conditions

STZ was initially discovered for its ability to induce diabetes in experimental animals, and it has been widely used to model both type 1 and type 2 diabetes in rodents. In type 1 diabetes, STZ is used to selectively destroy the insulin-producing beta cells of the pancreas, leading to insulin deficiency and hyperglycemia [144]. This model is commonly employed to study the pathophysiology of autoimmune diabetes and to test potential therapeutic interventions aimed at preserving beta cell function or restoring glycemic index. STZ has often been combined with a high-fat diet to model the induction of insulin resistance and beta cell dysfunction that leads to impaired glucose tolerance and hyperglycemia in models of type 2 diabetes. This model is rather close to the progressive nature of type 2 diabetes in humans and offers great value in the study of metabolic and molecular mechanisms underlying insulin resistance, obesity, and associated complications [145]. Now, in the current arena, ICV-STZ injections are an important part of developing an effective AD model in rodents. STZ is widely used to induce neurotoxicity and cognitive impairment similar to those of AD pathology in experimental animals. The optimum dose of STZ depends on the species, strain, age, and sex of the animals, as well as the intended extent and duration of cognitive deficits to be induced. Additionally, both the method of administration and the time of injections relative to the beginning of AD-like pathology may also modulate the efficiency and reproducibility of the mode. Nevertheless, it is essential to emphasize that neither of these factors nor dosing techniques effectively generates mature amyloid plaques or NFTs in this model. Thus, while dosage optimization helps produce comparable early-stage pathology, the fundamental constraint of the model in duplicating late-stage AD characteristics remains unaltered.

## Dosing patterns of streptozotocin

The dose of ICV-STZ is an important factor in simulating sAD in rodents, determining both the amount of neurodegeneration and the repeatability of cognitive abnormalities. Numerous studies have investigated the dose-response effects of STZ, revealing precise doses that cause cognitive deficits while avoiding severe neurological damage or systemic toxicity. Doses ranging from 1 to 3 mg/kg, delivered bilaterally on alternate days (e.g., Day 1 and Day 3), are thought to be ideal for producing progressive, AD-like characteristics while preserving animal survival and experimental reliability [146, 147]. Comparative studies have revealed dramatic differences between the peripheral and central delivery of STZ. ICV injections limit cerebral utilization of glucose without inducing peripheral hyperglycemia, whereas IP injections primarily target pancreatic β-cells to produce diabetes by hyperglycemia. This cerebral metabolic disturbance is associated with cognitive deficits, implying a significance for brain insulin resistance in STZ-induced neurotoxicity [148]. Higher STZ dosages resulted in early sporadic AD-like pathology, such as neuropathology, involving increased expression of GFAP, phosphorylated tau, APP, and Aβ aggregation [149]. These findings validate the dose-dependent degree of pathology and promote the use of modest, fractionated doses to imitate early-stage AD [21]. Several preclinical methods were evaluated in STZ-based models to confirm therapeutic relevance.

Earlier reported studies also demonstrated that Crocins from *Crocus sativus* L., for example, effectively restored STZ-induced memory deficits when administered at a dosage of 3 mg/kg on days 1 and 3. Exendin-4, a GLP-1 receptor agonist, enhanced memory and decreased tau hyperphosphorylation and GSK-3β expression in ICV-STZ rats, highlighting the model’s potential in testing AD therapies, particularly with concomitant type 2 diabetes [150]. In metabolic evaluations, STZ at dosages of 1–3 mg/kg caused significant decreases in brain glucose consumption, ATP content, and phosphocreatine levels, as well as cognitive dysfunctions similar to the pathology of AD [151]. These alterations were linked to increased Aβ1–42 accumulation, hyperphosphorylated tau, impaired mitochondrial function, and decreased brain weight, notably in the hippocampus and cortical areas in early sporadic AD-like pathology. Additional mechanistic investigations have investigated downstream effects of ICV-STZ, such as enhanced AChE activity and neuroinflammation. COX-1/COX-2 inhibitors lowered AChE activity while restoring cognitive performance, underlining the cholinergic and inflammatory networks involved in STZ-induced neurodegeneration [152]. ICV-STZ can cause memory impairments and increased Aβ and neurofilament proteins even 21 days after injection, according to temporal research. These alterations were distinct from locomotor defects, indicating cognitive impairments associated with AD [153]. Importantly, sex- and time-dependent differences in glucose metabolism and astrocyte function have been seen following STZ administration, indicating the necessity for individualized dosage procedures across diverse animal species [154]. Strategies that included androgen receptor inhibitors, daidzein, and galantamine-loaded hydrogels showed protective benefits in ICV-STZ rats by reducing oxidative stress, tau phosphorylation, and learning ability [149, 155, 156]. The variability in STZ dose (usually 1–3 mg/kg, bilaterally or unilaterally) has a significant influence on the onset, degree of severity, and type of neuropathological alterations seen. Initial studies used 3 mg/kg ICV dosages, resulting in significant neuronal loss and oxidative stress [157]. However, this high dosage is associated with acute neurotoxicity and decreased survival, making it unsuitable for studying chronic, progressive AD-like characteristics.

Later studies have shown that a lower dose (e.g., 1.5 mg/kg) injected in a split regimen (particularly in 3 mg/kg total, divided over two injections, 48-hour interval) causes more pronounced cognitive impairment, synaptic abnormalities, and insulin signaling disruption while preserving animal mortality [152, 158]. Such a split-dose paradigm is currently thought to be more trustworthy for mimicking progressive neurodegeneration, allowing for a more accurate assessment of therapeutic approaches [159, 160]. Nonetheless, dose-dependent thresholds are poorly defined, and inter-study heterogeneity exists due to changes in age, gender, and animal strain employed. Furthermore, very low doses may fail to elicit distinctive characteristics, including tau hyperphosphorylation or substantial amyloidogenesis, whereas extremely high doses may cause confusing disease due to widespread cytotoxicity [161, 162]. The reviewed data demonstrate that both the dosage and delivery schedule of ICV-STZ are critical in defining the degree of severity, recurrence, and translational significance of AD-like disease in animal models. A dose of 3 mg/kg applied bilaterally on alternate days appears to be the most reliable way for reproducing cognitive loss, neuroinflammation, tauopathy, and altered glucose metabolism without causing overt toxicity. This tailored dosage methodology is critical for mechanistic research and preclinical studies of neuroprotective treatments for sAD.

Animal-related parameters such as species, strain, sex, and age have a significant impact on the diversity of STZ-induced intracerebroventricular pathology [28, 163]. Rats, particularly the Wistar and Sprague Dawley species, show higher repeatability and more severe cognitive and histopathological deficits than mice at equal per-kg doses [1,4]. Aged animals are more susceptible to STZ-induced oxidative and insulin-signaling disruptions [164, 165], while younger cohorts may require a slightly greater cumulative dosage to produce equivalent impairments. Gender variations are also documented, with male rats exhibiting more cholinergic and metabolic abnormalities [166]. As a result, dosage optimization must take into consideration these biological characteristics in order to achieve translational validity. According to comparative assessments, the 3 mg/kg total cumulative dosage applied as 1.5 mg/kg bilateral ICV on days 1 and 3 results in the most persistent onset of sAD-like neurodegeneration in rats and mice, while maintaining neurotoxicity and mortality rates [167–169].

## Various methods of administering streptozotocin at different dosages

**Fig. 4:**
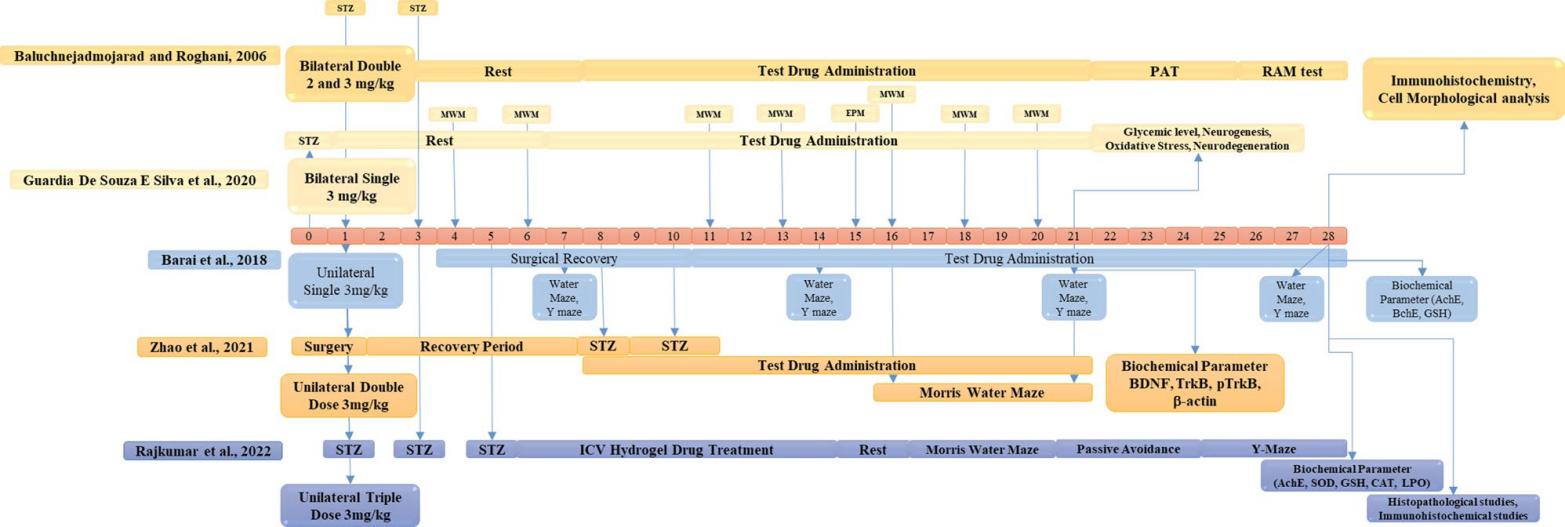
Various methods of administering streptozotocin at different dosages

In terms of dosing regimens, STZ can be administered in ways deemed appropriate for the experimental requirements. These are unilateral single, unilateral multiple, bilateral single, and bilateral multiple administrations. All of these regimes have their benefits and are chosen based on the aims of the research and the characteristics of the wanted AD model Fig. 4.

### Unilateral single-dose streptozotocin

Several studies have investigated the ability of a single unilateral ICV treatment of STZ to resemble sAD pathophysiology in rats. In a previous investigation, researchers evaluated the neuroprotective benefits of *Bergenia ciliata* against STZ-induced cognitive impairments at the 3 mg/kg dose. In this investigation, STZ treatment dramatically reduced learning and memory i rats, as evidenced by poor performance on the Morris Water Maze (MWM) and Y-maze tests. Interestingly, dose-dependent treatment of *Bergenia ciliata* extract ameliorated these deficiencies. It regulated cholinergic transmission, decreased oxidative stress, and maintained histological brain integrity [170]. In another investigation, the potential therapeutic benefits of geniposide were assessed using a similar paradigm. Unilateral 3 mg/kg STZ treatment contributed to tau hyperphosphorylation and neurofibrillary tangle development, which are early indicators of AD pathology. Geniposide therapy prevented these neurodegenerative alterations and increased spatial learning skills, most likely by activating the GLP1 receptor and modulating the PI3K/GSK3 pathway. Its excellent pharmacokinetic profile strengthens its translational usefulness [171].

### Unilateral double-dose streptozotocin administration

To develop a more persistent model of AD, researchers used a double-dose unilateral ICV injection of STZ (3 mg/kg on days 8 and 10). Earlier data also demonstrated that administration using sporoderm-removed Ganoderma lucidum spores at 360 and 720 mg/kg improved memory performance, mitigated STZ-induced elevations in Aβ and phosphorylated Tau (Ser199, Ser202, Ser396), and lowered BDNF and TrkB signaling pathways [172]. Similarly, Bergenin was examined with the same STZ dosage. Its treatment considerably enhanced behavioral performance while decreasing AChE and BuChE activity and raising GSH levels. Bergenin at 80 mg/kg significantly lowered Aβ-1–42 and p-tau levels, possibly through its antioxidant and anti-inflammatory properties [173].

### Unilateral triple-dose streptozotocin

The researcher studied different doses of STZ for ICV administration to induce AD-like symptoms. However, significant AD-like symptom induction was not observed with unilateral single or double administrations. Therefore, the scientist explored another approach involving triple ICV administrations to enhance AD-like symptom induction. The researcher explored the effectiveness of a galantamine-tethered hydrogel as a new therapeutic approach for managing STZ-induced AD in Wistar rats. They administered a dose of 3 mg/kg of STZ on days 1, 3, and 5 via the ICV route and evaluated the potential benefits of the galantamine-tethered hydrogel. The findings suggest that hydrogel-based galantamine drugs effectively reduce Aβ aggregation, enhance the neuroinflammatory response, boost antioxidant activity, and promote neuronal growth [174].

### Bilateral single-dose streptozotocin

The previously reported data demonstrated that bilateral ICV administration of STZ at 2 mg/kg caused deficits in memory and tau/Aβ pathology associated with early sporadic AD-like pathology. Treatments with anandamide (AEA) reduced these effects, enhancing object recognition skills and reducing ventricular hypertrophy. This lends credence to the efficacy of cannabinoid-based therapy in AD [175]. Other investigations found that administering royal jelly orally after STZ (3 mg/kg) increased hippocampal activity, neurogenesis, and memory retention [176]. Betulinic Acid (1 µM, intra-hippocampally) reduced cognitive impairment and synaptic activity in STZ-injected rats [177]. Ectoine significantly improved neuroprotection by affecting the expression of Synaptophysin-A, MAPK-3, TNF-α, and VEGF in the hippocampus. In contrast, varying STZ doses (1–3 mg/kg) revealed that higher doses triggered severe neurodegeneration, while lower doses led to delayed but progressive cognitive deficits. These results point to a dose-dependent and time-sensitive model of AD [147].

### Bilateral double-dose streptozotocin

In earlier findings, to examine neurological and respiratory impairment, bilateral STZ dosages of 2 and 3 mg/kg were delivered. Both caused considerable hippocampal shrinkage and gliosis, especially in the nTS area, indicating site-specific disease [178]. Furthermore, data also evidence that naringenin at 50 mg/kg/day for three weeks significantly improved learning, memory, and oxidative imbalance in rats following 3 mg/kg STZ injections on days 1 and 3 [27]. Trans-resveratrol had comparable cognitive rescue benefits, increasing memory and reducing oxidative damage, as seen by lower MDA and intact glutathione levels [179]. Tetramethylpyrazine inhibited GSK-3β, therefore preventing bilateral STZ-induced memory impairment [180]. Its effects were similar to those reported across both transgenic and non-transgenic AD mouse models, highlighting the ICV-STZ model’s translational prospects.

### Bilateral triple-dose streptozotocin

The researcher administered STZ via ICV to create an AD model in aged and experienced rats. They divided the total dose of 4.5 mg/kg STZ into three equal doses of 1.5 mg/kg each, given on days 1, 3, and 5, with the STZ dissolved in a 0.05 M citrate buffer. The study observed both cognitive and non-cognitive behaviors following ICV-STZ administration in these rats. While STZ treatment led to increased novelty-induced exploratory activity in the open-field test, it did not significantly affect anxiety levels in the elevated plus maze (EPM). Additionally, biochemical markers such as hippocampal β-amyloid and phospho-tau levels did not exhibit significant differences [158].

Previous reported data suggested the various unilateral and bilateral ICV-STZ dosing strategies that underscores the versatility and sensitivity this model in replicating multiple hallmarks of sAD. Cognitive impairments, oxidative stress, tau hyperphosphorylation, Aβ buildup, neuroinflammation, and synaptic abnormalities, pathophysiological characteristics resembling early to advanced stages of AD are successfully induced by single or recurrent STZ injections, depending on dosage. Furthermore, numerous natural and synthetic agents, including bergenia, geniposide, royal jelly, ganoderma spores, and trans-resveratrol, demonstrated promising neuroprotective effects by targeting diverse pathways such as cholinergic neurotransmission, PI3K/GSK-3 signaling, oxidative balance, and neurogenesis. These findings validate the relevance of the ICV-STZ model and highlight its utility in screening potential therapeutic agents for sAD.

## Approaches for testing drug dosages in STZ-induced Alzheimer’s disease models

In the current scenario, researchers face significant hurdles in developing novel therapies for AD due to the intricate nature of its underlying mechanisms. In response to this challenge, researchers have shifted their focus towards utilizing animal models of AD to gain deeper insights into disease progression and to assess potential therapeutic strategies. Among these models, STZ-induced AD models have emerged as prominent tools, as they successfully mimic critical aspects of AD pathology. In this scenario, evaluating the dosages of drugs in STZ-induced AD models has evolved into a pivotal phase within preclinical drug development. This methodology enables researchers to analyze the effectiveness, safety, and ideal dosage levels of potential drugs before progressing to human clinical trials Table 1.

**Table 1:** Comparison of different administration schedules for streptozotocin

Experimental Animal Models	Experimental Animals	STZ Administration Schedule	Site of Administration	Dose of STZ	Targeted Brain Location	Time points post-STZ injection	Pathological and Cellular Outcomes	Physiological Impacts	References
Model of AD	Rats	Single Administration of STZ	Unilateral	3 mg/kg in citrate buffer	Lateral Ventricles, and the hippocampus	7 days	Modulating cholinergic activity, reducing oxidative stress, and preserving histopathological integrity	Cognitive deficits	[170, 171]
Model of AD (STZ-induced AD is site-specific, particularly in the caudal/intermediate nTS region)	Rats	Double Administration (days 1 and 3)	Bilateral	2 and 3 mg/kg	Caudal/intermediate nTS region	21 days	Synaptic loss and gliosis in the nucleus tractus solitarii (nTS), reduction in synaptic density.	Significant hippocampal atrophy, exhibited respiratory dysfunction, astroglial and microglial activation.	[178, 181]
Alzheimer’s Disease Like Sporadic Dementia	Rats	Double Administration (days 1 and 3)	Unilateral	2 mg/kg	Hippocampus	5 days	Ventricle enlargement, which is indicative of neuronal loss	Cognitive impairment and synaptic dysfunction	[138, 163]
sporadic Alzheimer’s disease	Rats	Double Administration (days 1 and 3)	Unilateral	3 mg/kg in aCSF	Hippocampus	7 days	Abnormalities in the hippocampal long-term potentiation (LTP)	Increase Aβ expression and Tau protein expression and phosphorylation at Ser199, Ser202, and Ser396, decreased neurotrophic factors, including BDNF and TrkB,	[172, 182, 183]
AD model	Rats	Triple Administration (days 1, 3, and 5)	Unilateral	3 mg/kg in citrate buffer	Cortex and Hippocampus	5 days	Deterioration of learning and memory, neuroprotective defects, and amyloid plaque formation in the brain	Apoptosis in neurons is characterized by damaged neurons and amyloid plaque formation	[121, 174]
AD model in a translationally relevant, aged, and experienced rat population	Rats	Triple Administration on days 1, 3, and 5,	Bilateral	4.5 mg/kg STZ into three equal doses in citrate buffer	Hippocampus	14 days	Significant memory Loss	Impairments in spatial and recognition memory but not in fear learning/memory, visual discrimination, and social learning; however, it induced impulsive-like behavior. The β-amyloid level was not increased, probably because of the high basal level.	[184, 185]
sAD model	Rats	Single Administration of STZ	Bilateral	3.0 mg/kg	Hippocampus	7 days	Declining neurons in the hippocampus, oxidative stress	Insulin resistance and promoting neurogenesis	[183, 186]
Anxiety-Like Behaviors in Streptozotocin-Induced Diabetes	Mice	Single Administration of STZ	Intraperitoneal	150 mg/kg	Glial cells in the hippocampus, astrocytes, and oligodendrocytes	7 days	Dysregulation of the hypothalamic-pituitary-adrenal axis (HPA), which can trigger insulin resistance	Anxiety performances	[187]
Anxiety-Like Behaviors in Streptozotocin-Induced Diabetes	Rat	Single Administration of STZ	Intraperitoneal	50 mg/kg	Whole Brain	3 days	Increased noradrenergic and serotonergic activity	Anxiety-like Behaviour	[187, 188]
Depressive-like behavior in Streptozotocin-Induced Diabetic rats	Rat	Single Administration of STZ	Intraperitoneal	30 and 60 mg/kg	Whole Brain	14 days	Alteration of dopamine and serotonergic systems	Depressive-like behaviour	[189, 190]
Anxiolytic and antidepressant behavior in STZ-induced diabetic rats	Rat	Single Administration of STZ	Intraperitoneal	60 mg/kg	Prefrontal cortex hippocampal	3 days	Decrease in SOD, CAT, GSH, and total antioxidant capacity, NF-κB, IL-6, and IL-1β are elevated.	Astrocyte activation	[191, 192]
sAD model	Rat	Double (days 1 and 3)	Unilateral ICV	2 mg/kg	Hippocampus	5 days	Ventricle enlargement, synaptic dysfunction	Impaired learning and memory	[28]
AD model	Rat	Double (days 1, 3)	Unilateral ICV	3 mg/kg in citrate buffer	Subventricular zone (SVZ) and dentate gyrus (DG)	21 days	Glial fibrillary acidic protein (GFAP) & nuclear factor kappa-light-chain-enhancer of activated B cells (NFκB), and diminished insulin signaling	(5-Bromo-2’−deoxyuridine) BrdU+ Nestin+ cells, Doublecortin (DCX+) cells and BrdU+ NeuN+ cells	[129]
sAD model	Rat	Double (days 1, 3)	Unilateral ICV	3 mg/kg	Hippocampus	14 days	Glial fibrillary acidic protein (GFAP), Nrf2/ARE pathway	Cognitive dysfunction	[193]
Site-specific AD model (nTS region)	Rat	Double (days 1 and 3)	Bilateral ICV	3 mg/kg	Caudal/intermediate nTS	21 days	Synaptic loss, gliosis, and microglial activation	Respiratory and hippocampal dysfunction	[194]
sAD model	Rat	Double (days 1 and 3)	Unilateral ICV	3 mg/kg in aCSF	Hippocampus	7 days	↑Aβ, ↑Tau (Ser199/202/396), ↓BDNF, ↓TrkB	Cognitive decline, poor retention	[19]

### Effect of test drug administration for 14 days

Previous studies on ICV-STZ-induced AD the 2-week treatment with tetramethyl pyrazine rescued both short-term and long-term fear memory impairments induced by ICV injection of STZ in the well-known AD rat model. Further presented data demonstrated that mice treated with TMP develop the opposite pattern of the STZ-induced impairments in the inhibitory avoidance tasks and the Morris water maze task, a normal function in which the hippocampus, a main brain region for learning and memory [195]. Also, researchers investigated the therapeutic potential of the PDE-4 inhibitor roflumilast against ICV-STZ-induced AD-like symptoms in rats. After a 7-day surgical recovery, roflumilast 0.51 mg/kg was delivered orally from day 7 through day 21 of the study [196] Table 2.

**Table 2:** Quantitative summary of ICV-STZ dose regimens and outcomes in AD models

Dose (mg/kg) & Regimen	Cognitive Deficit Onset (days)	Behavioural Performance	Biochemical Makers	Molecular Pathways	Mortality (%)	Onset/severity of cognitive deficits	References
3 mg/kg, 1 and 3 days	14 days	Morris Water Maze	TBARS, GSH, GR, CAT, COX-2, IL-8	GFAP, iNOS	0	Optimal mimic of sAD	[197]
3 mg/kg, 1 and 3 days	14 days	Passive avoidance, and Morris Water Maze	TBARS, GSH, GR, GSSG, ChAT, MDA	4-hydroxynonenal (4-HNE)	Not reported	Sporadic dementia of Alzheimer’s type (SDAT)	[80]
35 mg/kg/i.p	28 days	Morris Water Maze	GSH, SOD, CAT, MDA	α7 nicotinic receptors	0	Diabetes-induced Alzheimer’s disease-like phenotype	[198]
3 mg/kg, 1 and 3 days	14 days	Morris Water Maze	GFAP, β-secretase	Nrf2/ARE pathway	Not reported	Optimal mimic of sAD	[193]
3 mg/kg, 1 and 3 days	14 days	Passive avoidance paradigm, and the Elevated plus maze	ChAT	Cholinesterase activity	0	Optimal mimic of sAD	[199]
3 mg/kg, day 1	28 days	Morris Water Maze	TLR4, RAGE, TNF-α, and NF-κB	methylglyoxal/ RAGE/NOX‑2	Not reported	Optimal mimic of sAD	[200]
3 mg/kg, 1 and 3 days	26 days	Morris Water Maze	Nitrite/nitrate and TBARS	BDNF expression	0	Optimal mimic of sAD	[201]

### Effect of test drug administration for 21 days

In the study, researchers investigated the protective effects of evodiamine in an ICV-STZ-induced AD model. They administered evodiamine 50 or 100 mg/kg/day, orally to animals from day 1 to day 21 of the study, and found improvements in STZ-induced cognitive deficits using Novel Object Recognition and Morris Water Maze tests. Evodiamine significantly attenuated the STZ-induced increase in AChE activity and MDA levels [202]. The researchers also looked into the neuroprotective effects of 7, 8-dihydroxyflavone after oral administration in the ICV-STZ-induced AD mouse model. In the study, 7, 8-dihydroxyflavone was administered orally from the beginning of the protocol until the behavioral study on day 21. According to the results, the treatment significantly improved ICV-STZ-induced cognitive impairment during both the Morris Water Maze and Novel Object Recognition tests. Also, restoration of GSH, catalase, SOD, GPX, LPO, PCO, and nitrite levels in the cortex and hippocampus regions of the brain was observed [203].

### Effect of test drug administration for 18 days

In an earlier reported study, the researchers investigated the role of Pseudoginsenoside-F11 on cognitive dysfunction and tau hyperphosphorylation in the early sporadic AD-like pathology rat model induced by ICV administration of STZ. In this study, the PF-11 administration was initiated from the 3^rd^ day of STZ administration; the dosing range for the same was 2–8 mg/kg till day 21. They showed that STZ ICV infusion had a significant effect on cognitive function, tau phosphorylation, and the insulin signaling pathway in the hippocampus. Further, the STZ-ICV infusion revealed the calpain I/cyclin-dependent protein kinase 5 (CDK5) signaling pathway to be significantly upregulated in the hippocampus. The PF11 treatment reduced neuronal loss, protected the structure of the synapse, and modulated the STZ-induced expression of tau phosphorylation in the hippocampus via the insulin signaling pathway and the calpain I/CDK5 signaling pathway [204].

### Effect of test drug administration for 11 days

Moreover, the researchers investigated the neuroprotective effects of voglibose in an ICV-STZ-induced animal model of AD. Voglibose administration at a dose of 10, 25, and 50 mg/kg began on day 3 following STZ administration and continued until day 14, totalling an 11-day treatment period. In their research, they conclude that in comparison to ICV-STZ-treated rats, voglibose treatment at doses of 10, 25, and 50 mg/kg significantly reduced acetylcholinesterase (AChE) and malondialdehyde (MDA) activities while enhancing antioxidant enzyme activities. Molecular analyses indicated significant reductions in TNF-α, IL-1β, and CRP activity, along with a notable decrease in Aβ aggregation demonstrated by western blot results. Histopathological assessments revealed a substantial improvement in cellular morphology, including clear cytoplasm and healthy neuronal cells, following voglibose treatment [205].

The investigation of dosages of drugs in STZ-induced AD models is a vital step in improving therapeutic discoveries against this complicated neurodegenerative condition. The studies stated show that altering the duration and dosage of test medication administrations, from 11 to 21 days, can dramatically improve cognitive impairments, metabolic imbalances, neuroinflammation, and structural degradation associated with AD pathology. Tetramethyl pyrazine, roflumilast, evodiamine, 7,8-dihydroxyflavone, Pseudoginsenoside-F11, and voglibose all show promising therapeutic profiles by targeting multiple pathological hallmarks such as oxidative stress, tau hyperphosphorylation, mitochondrial dysfunction, and glial activation. These findings emphasize the importance of selective dosage regimes and treatment timeframes in maximizing neuroprotective benefits.

## Dose variation of STZ for different diseases

### Anxiety-like behavior in streptozotocin-induced diabetes

Recent studies have highlighted the manifestation of anxiety-like behaviors and cognitive dysfunction in streptozotocin (STZ)-induced diabetic models, attributed primarily to neuroinflammatory mechanisms. In one investigation, researchers administered a single intraperitoneal dose of STZ (150 mg/kg) to mice to induce diabetes-associated anxiety-like phenotypes. The results demonstrated significant impairments in memory and cognitive function, suggesting that anxiety and memory deficits are key neuropathological features in STZ-induced diabetic mice. Notably, glial fibrillary acidic protein (GFAP), a marker of astrocyte activation, was significantly upregulated at the protein level, indicating pronounced neuroinflammation within the central nervous system. Moreover, elevated levels of the pro-inflammatory cytokine interleukin-6 (IL-6) were detected in brain tissue samples. Behavioral analyses revealed that diabetic mice exhibited reduced locomotor activity and decreased exploration of the central zone in the open field test, further supporting the presence of anxiety-like behaviors [206].

In another study, anxiety-like behavior was evaluated in STZ-induced diabetic rats. STZ was administered intraperitoneally at a dose of 50 mg/kg, dissolved in citrate buffer (pH 4.5), and behavioral assessments were conducted 72 hours post-injection. The anxiolytic effects of diazepam were also investigated. Findings demonstrated that diabetic rats exhibited significantly reduced rearing and central locomotor activity in the open field test, as well as decreased entries into both the open and closed arms of the elevated plus maze, with an increased preference for the closed arms indicates the elevated anxiety levels. Biochemical analyses revealed a significant increase in brain tribulin activity, an endogenous marker associated with anxiety via inhibition of monoamine oxidase A (MAO-A). These results collectively suggest that STZ-induced diabetes markedly enhances anxiogenic behaviors in rodents compared to non-diabetic controls [207].

### Depressive-like behaviour in streptozotocin-induced diabetic rats

The researchers investigate the depressive behavior of STZ-induced diabetes, characterized by hyperglycemia. STZ causes a state of absolute or relative insulin deficiency, which is the major risk factor for depression in patients with diabetes. STZ is administered at a dose of 30 and 60 mg/kg body weight via the intraperitoneal route for the induction of diabetes and depression in rats. STZ has depressive-like behavior two weeks after the injection. Results postulated that STZ has been reported to increase the immobility time, which indicates the depressive-like behavior that is caused by STZ. The interplay between dopamine and serotonergic systems can further affect mood, as an imbalance in one system affects the other, possibly leading to depression [189]. In another study, researchers investigated the antidepressant-like effect of Zamzam water on STZ-induced diabetic rats. STZ at the dose of 60 mg/kg was dissolved in citrate buffer, inducing type 1 diabetes mellitus. Anxiety-depression-like behavior was assessed in rats induced with diabetes. The diabetic rate reduced the immobility in the FST and anxiety-like behavior, as indicated by the elevated percentage of open field entries and time in EPMT. The animal remained for some time in the central zone, but remained in closed arms in the EPMT. It decreases SOD, CAT, GSH, and total antioxidant capacity in the diabetic rat and increases the histological structures preserved in the prefrontal cortex and hippocampus, NFκB, IL-6, and IL-1β, which are pro-inflammatory mediators. It also increases, in the prefrontal cortex and the hippocampus, TNF-α and GFAP immunoexpression [191].

## Stereotaxic coordinates and intracerebroventricular injection procedure

In order to ensure anatomical precision, ICV administrations were carried out using stereotaxic coordinates that were precisely obtained from Paxinos and colleagues’ conventional brain atlases. The Paxinos & Watson Rat Brain Atlas was used to target the lateral ventricle in adult Wistar and Sprague Dawley rats, with coordinates at anteroposterior (AP) −0.8 to −1.0 mm, mediolateral (ML) ±1.4–1.6 mm, and dorsoventral (DV) −3.5 to −4.0 mm from bregma [208, 209]. These coordinates offer dependable ventricular entrance in adult rats and are often employed in reported ICV-STZ and cerebral infusion research. According to the Franklin & Paxinos Mouse Brain Atlas, the ventricles of adult C57BL/6 mice were usually located at AP −0.3 mm (±0.3 mm range), ML ±1.0–1.2 mm, and DV −2.5 to −3.0 mm [210, 211]. These species-specific coordinates are similar throughout mouse ICV infusion guides (e.g., ALZET recommendations) and represent known anatomical distinctions across rodents [212]. To prevent reflux and tissue damage, injection quantities were administered at 0.5–2 µL/min in accordance with conventional neuropharmacological procedures, with 5–10 µL per ventricle in rats and 2–5 µL in mice. To reduce mechanical damage, a Hamilton 5–10 µL microsyringe with a 26–30 G needle was utilized. As advised by ventricular infusion validation guidelines, placement accuracy was verified in pilot animals using Evans Blue or Fast Green dye. Following accepted neurosurgery protocols, stereotaxic procedures were carried out utilizing a Kopf or Stoelting frame with a calibrated microinfusion device for regulated administration. In accordance with institutional animal ethical regulations, post-operative care involved thermal support, regular analgesia (buprenorphine or meloxicam), and daily weight and neurological activity monitoring [213].

## Validity of the ICV-STZ model in Alzheimer’s disease research

Predictive validity is a laboratory model’s ability to properly predict the effectiveness of therapeutic drugs in human clinical situations. In the scenario of AD, an experiment with good predictive validity must adapt to pharmacological therapies in the same way as human patients would. The ICV-STZ paradigm has been useful in assessing several treatment medications for AD-related diseases. For example, studies have shown that administering insulin stimulants, like pioglitazone, improves cognitive impairments and lowers neuroinflammation in ICV-STZ-treated animals, reflecting clinical findings in AD patients [133]. Similarly, cholinesterase inhibitors, such as donepezil, have been demonstrated to enhance memory retention and preserve cholinergic function in this model, which is consistent with their clinical success in treating AD symptoms [214]. Furthermore, antioxidants such as resveratrol have demonstrated neuroprotective benefits in ICV-STZ-induced animals by reducing oxidative stress and improving cognitive performance, which is consistent with findings from human investigations [179]. These pharmacological reactions demonstrate the model’s effectiveness in predicting treatment results. However, despite the ICV-STZ model mimicking key elements of sAD, such as resistance to insulin and memory loss, it does not fully account for the illness’s amyloid-beta plaque development. As a result, the model’s predictive value for amyloidogenesis-targeted medicines may be restricted. In conclusion, the ICV-STZ model has high predictive validity, especially for therapies targeting metabolic disorders, oxidative damage, and cholinergic deficiencies. Its procedures for multiple pharmacological treatments give important information about prospective therapy options for sAD.

## Limitations of the ICV-STZ model in Alzheimer’s disease research

The ICV-STZ has been widely used as a non-transgenic model for studying sAD. This model accurately mimics numerous essential aspects of AD, including cognitive impairments, central resistance to insulin, oxidative stress, mitochondrial failure, and neuroinflammation. These pathogenic results make it a great tool for studying the metabolic and proinflammatory components of AD, particularly within the context of the “type 3 diabetes” paradigm [28, 76]. Despite these benefits, there are certain restrictions and issues to consider. The ICV-STZ paradigm leads to persistent soluble Aβ elevation, tau hyperphosphorylation, decreased insulin signaling, and neuroinflammation, but lacks the classic neuropathological hallmarks of AD pathology, including substantial amyloid-beta plaque and mature NFT accumulation. As a result, this model is most effective as a portrayal of sAD-like metabolic and molecular abnormalities rather than an ideal replication of all histopathological hallmarks of the disease. Furthermore, full Aβ plaque accumulation and NFT generation are unavailable, particularly when compared to genetically transgenic animals. This distinction is critical for correctly interpreting the model, which adequately reflects the physiological, inflammatory, and synaptic impairments associated with early sAD but has many drawbacks when examining progressive plaque or tangle disease. As a result, certain investigators claim that this model represents persistent neuroinflammation or metabolic encephalopathy instead of a full model of AD [18, 76]. Furthermore, the disease development in this model is extremely rapid and experimentally caused through direct intracerebral injection, which differs dramatically from the slow and multifaceted beginning of AD in individuals. This raises concerns about its applicability to clinical settings, notably about long-term neurotoxicity and age-related disease [28, 198]. A further important restriction is diversity in technique among studies, such as changes in STZ dose, treatment frequency, and animal strain, that can result in conflicting results and impair repeatability. For example, studies have demonstrated that the effects of ICV-STZ differ between strains and rely on the age of rodents and experience level, emphasizing the requirement for standard procedures to improve the universal validity of the model [198]. In addition, while the ICV-STZ paradigm causes some AD-like characteristics, it does not reliably mimic other elements of the illness. Some investigations have found that the model may not accurately simulate the cholinergic impairments seen in AD, especially over longer periods [21]. Furthermore, the exact process by which STZ causes these pathogenic alterations remains unknown, prompting additional studies to unravel the fundamental processes [215]. Nonetheless, when understood within its boundaries, the ICV-STZ model remains a useful and affordable experimental tool for studying the metabolic and proinflammatory aspects of AD. A thorough assessment of its limits is required for adequate study design and reaching relevant findings on its clinical application.

## Discussion

Considering the rapid expansion of AD models, the ICV-STZ model remains relevant due to its capacity to replicate sAD-like pathology, specifically insulin resistance, oxidative stress, neuroinflammation, and cognitive impairments. These features are more consistent with the metabolic and sporadic characteristics of late-onset AD than several genetically engineered models, which mostly depict familial variants. As the research attempts to achieve greater translational accuracy, the ICV-STZ model stands out for its distinctive ability to recapitulate numerous disease aspects within a reasonable experimental framework. Inconsistencies in dosage selection, injection procedures, and inadequately described molecular consequences continue to hinder reliability and translational interpretation. Although the ICV-STZ model has been widely employed, variations in dosage techniques (varying from 1 mg/kg to 3 mg/kg), number of injections (single versus double), and chronological interval between doses have resulted in conflicting findings across investigations. This diversity makes it challenging to compare results and draw general conclusions.

Furthermore, whereas cognitive and behavioral results are often reported, the underlying molecular markers, such as changes in insulin receptor signaling, mitochondrial function, and inflammatory cascades, are not consistently documented or standardized across research. Dose-dependent disparities in neurotoxicity and behavioral consequences demand an improved comprehension of threshold and saturation impacts, particularly when comparing the early and late stages of AD. The impact of single vs bilateral injections and the timing of dosages is not well-documented in terms of progression dynamics or molecular modifications, presenting a gap in protocol standardization. The ICV-STZ model can help us comprehend the sAD, which accounts for a significant number of occurrences in humans. Nevertheless, differences in experimental techniques have resulted in conflicting findings among investigations. This study emphasizes the need to standardize factors such as dose and delivery regimens to improve the repeatability of outcomes. ICV-STZ injections are used to imitate sporadic AD in animal models by generating cognitive impairments and neurodegenerative alterations reminiscent of those seen in human AD.

The regimen of STZ delivery is critical in determining the model’s repeatability. A bilateral ICV method assures an even distribution of STZ throughout the brain ventricles than unilateral administration, which can result in asymmetric pathology and variable effects [216]. The time between injections also impacts disease development. A standard technique combines two 1.5 mg/kg injections on days 1 and 3, which reduces acute toxicity while encouraging long-term neurotoxicity [19, 217]. Single-dose regimens, while easier, can result in uneven phenotypes, notably delayed cognitive impairments and metabolic impairment [218]. In addition, the vehicle and solvent employed for STZ delivery (often citrate buffer at pH 4.5) might influence compound stability and eventual neurotoxicity, but this component is frequently underreported or omitted. These differences underscore the critical need for consensus recommendations on administration techniques to increase cross-study comparability and translational utility. Based on previous investigations, the most optimal doses for the experimental induction of AD-like pathology range from 1-3 mg/kg. Earlier findings also revealed that low doses of STZ are inconsistent in proving AD pathology. Whereas, higher doses significantly produce irreversible brain damage with notable toxicities and mortality in the experimental animals.

When comparing single-administration versus multiple injections with multiple low-dose STZ has been proven to be the optimal regimen and has a gradual onset of AD-like pathology compared to high-dose single administration. Furthermore, researchers have also demonstrated that multiple bilateral administrations of low doses of STZ have better validity in experimental models compared to single-dose unilateral administrations. Earlier investigations delved into various protocols for the standard duration of the regimen. The data suggested that the 21-day protocol is the most appropriate among all the reported regimens, which significantly induces AD-like pathology in rodents with prompt symptoms by utilizing STZ as an inducer. The studies also show that STZ-induced pathology primarily involves several cellular and biological processes such as oxidative stress, mitochondrial dysfunction, neuroinflammation, and insulin signaling pathology. The ICV-STZ model’s validity is frequently assessed based on its capacity to replicate important clinical, biochemical, and histological characteristics of sAD. It involves impaired spatial learning and memory, decreased glucose metabolism in the brain, elevated oxidative stress, and cholinergic dysfunction, all of which are associated with symptoms of AD and pathologies. Furthermore, ICV-STZ-treated rats show molecular changes such as tau hyperphosphorylation, amyloidogenic APP processing, insulin receptor resistance, and glial activation, suggesting construct validity. Face validity is further supported by the model’s progressive nature and susceptibility in certain brain regions, most notably the hippocampus and cortex. However, its predictive value warrants more investigation, particularly in assessing the pharmacological effects of disease-modifying drugs.

Nonetheless, it is crucial to note that the categorization of the ICV-STZ model as an AD model is still debatable. Several research groups contend that the pathogenic results of this model, including insulin signaling deficiencies, mitochondrial dysfunction, and neuroinflammation, are more closely related to elements of metabolic or neurodegenerative disorders in general than to AD specifically. This disparity in interpretation emphasizes the necessity for deeper comprehension and differentiation of the model, which may include multi-modal evaluations to establish its translational bounds. The impact of age, sex, and genetic makeup on STZ sensitivity needs to be examined, since these factors may explain discrepancies found in research. Another important aspect of this model is the temporal monitoring of disease development. Identifying the first molecular alterations after STZ treatment may provide insight into crucial processes that cause subsequent neurodegeneration. It is also vital to assess whether ICV-STZ models replicate non-cognitive symptoms of AD, including olfactory impairments, mood abnormalities, and glial activation.

Finally, integrating ICV-STZ with other risk factor models (for example, aging, a high-fat diet, or ecological stress) may increase the model’s ecological validity. It is unclear how these combinatorial techniques alter dosage thresholds and behavioral traits. These key concerns not only emphasize gaps in existing comprehension but also provide techniques for improving the ICV-STZ model’s value in identifying pathophysiological processes and evaluating potential treatment for sAD. Demonstrating the optimal dosage, administration, and duration of the regimen will prove game-changing for researchers, enabling them to replicate AD pathology and significantly improve the utilization of the STZ model in preclinical settings.

## Conclusions

The ICV-STZ administration is a widely used method for modeling the pathophysiological conditions of sAD, such as insulin resistance, oxidative stress, and neuroinflammation. However, major discrepancies in dosage regimens, administration schedules, and biochemical result reporting reduce the translational utility and repeatability. Based on the available information, we suggest that a dual bilateral ICV-STZ at 3 mg/kg dose (1.5 mg/kg) on days 1 and 3, with subsequent behavioral and biochemical assessments on days 14 and 21, is an optimal approach for developing consistent AD-like characteristics. Additionally, the model accurately reproduces critical clinical aspects such as tau pathology, cholinergic impairments, glial activation, and insulin-dependent signaling dysfunction. The critics claim that referring to the ICV-STZ AD model could simplify the unique processes of sAD, thereby bending treatment investigations. Instead, they suggest that it should be used as a model of insulin-resistant brain state or type 3 diabetes, with a focus on neuroinflammatory and metabolic abnormalities rather than conventional amyloid or tau pathology. ICV-STZ administration consistently increases soluble Aβ species and hyperphosphorylated tau builds up, indicating early AD-like biological abnormalities. Nevertheless, these changes frequently take place in the absence of extrinsic amyloid-beta plaque development or mature NFTs, suggesting that the model replicates early molecular disruptions rather than complete structural markers of AD. Nonetheless, there is ongoing disagreement over its specificity to AD vs other types of metabolic neurodegeneration, highlighting the importance of standardized procedures, chronological characterization, and multi-modal validation. Future modifications that incorporate risk variables like as aging or metabolic stress may improve the ecological and translational relevance of this model, hence increasing its value in preclinical AD research.

## Data Availability

Not applicable.

## Abbreviations

AD: Alzheimer’s disease
Asp2: Aspartyl protease 2
Aβ: Amyloid beta
BACE 1: Beta-secretase 1
CCL2: Chemokine (C-C motif) ligand 2
CDK5: Cyclin-dependent kinase-5
EphA: Ephrin type-A receptor 4
Fzd: Frizzled
ICV: Intracerebroventricular
IL-18: Interlukin-18
IL-1β: Interlukin-1β
IR: Insulin receptors
IRBS: Insulin-Resistant Brain State
LilrB2: Leukocyte immunoglobulin-like receptor B2
nAChR: Nicotinic acetylcholine receptor
NaKα3: Sodium-Potassium-ATPase Alpha 3 receptor
NFTs: Neurofibrillary tangles
NLRP3: Nucleotide-binding domain, leucine-rich–-containing family, and pyrin domain–-containing-3
PGRMC1: Progesterone receptor membrane component 1
PICALM: Phosphatidylinositol-binding clathrin assembly protein
PKB: Protein kinase B
STZ: Streptozotocin
TLRs: Toll-like receptors
